# Temporal Variations in the Effective Reproduction Number of the 2014 West Africa Ebola Outbreak

**DOI:** 10.1371/currents.outbreaks.9e4c4294ec8ce1adad283172b16bc908

**Published:** 2014-09-18

**Authors:** Sherry Towers, Oscar Patterson-Lomba, Carlos Castillo-Chavez

**Affiliations:** Mathematical and Computational Modeling Sciences Center, Arizona State University, Tempe, Arizona, USA; Department of Biostatistics, Harvard School of Public Health, Boston, Massachusetts, USA; Simon A Levin, Mathematical, Computational and Modeling Sciences Center, School of Human Evolution and Social Change, Arizona State University, Tempe, Arizona, USA

## Abstract

Background The rapidly evolving 2014 Ebola virus disease (EVD) outbreak in West Africa is the largest documented in history, both in terms of the number of people infected and in the geographic spread. The high morbidity and mortality have inspired response strategies to the outbreak at the individual, regional, and national levels. Methods to provide real-time assessment of changing transmission dynamics are critical to the understanding of how these adaptive intervention measures have affected the spread of the outbreak. Methods In this analysis, we use the time series of EVD cases in Guinea, Sierra Leone, and Liberia up to September 8, 2014, and employ novel methodology to estimate how the rate of exponential rise of new cases has changed over the outbreak using piecewise fits of exponential curves to the outbreak data. Results We find that for Liberia and Guinea, the effective reproduction number rose, rather than fell, around the time that the outbreak spread to densely populated cities, and enforced quarantine was imposed on several regions in the countries; this may indicate that enforced quarantine may not be an effective control measure. Conclusions If effective control measures are not put in place, and the current rate of exponential rise of new cases continues, we predict 4400 new Ebola cases in West Africa during the last half of the month of September, with an upper 95% confidence level of 6800 new cases.

## Introduction

Ebola virus disease (EVD) is a severe, often fatal viral infection in humans, with a case fatality risk (CFR) of up to 90% in previous outbreaks 1 ^,^ 2 ^,^ 3 ^,^ 13 ^,^ 14. There have been 13 recorded outbreaks since 2000, associated with at least two distinct strains of the Ebola virus (Ebola-Zaire and the Ebola-Sudan) 23, but the current West African outbreak has spread over the largest geographic area and caused the greatest number of infections and deaths 3 ^,^ 15 ^,^ 16 ^,^ 22 ^,^ 23. Critically, unlike previous epidemics, this outbreak has spread to densely populated areas, increasing the risk of international spread 22; the problem is compounded by lack of resources for effective quarantine and isolation in the under-developed countries that have been affected, and the high mobility of the population in a region with porous borders.

The spread of EVD requires direct contact with infected blood, tissue, or body fluids of either the living ill or the recently deceased, and the disease is particularly prone to transmission in unprotected homecare settings and during traditional burials 24 ^,^ 25. Symptoms typically appear after 2–21 days, and the disease quickly progresses and kills most infected patients within a few days 13 ^,^ 14. No licensed vaccine or specific treatment is currently available 26, leaving improved hygiene, quarantine, isolation, and social distancing as the only potential interventions. In an attempt to quell the outbreak, on August 1st the governments of Guinea, Liberia, and Sierra Leone announced plans to impose a military-enforced mass quarantine of entire regions and villages (referred to as *cordons sanitaire*), in an attempt to prevent spread of the disease to other areas 17. The spread of disease within the isolated areas has typically been allowed to run its course 19 ^,^ 20.

Assessing the net positive or negative impact of such attempted intervention measures involves assessment of a complex adaptive system, where the system dynamics are intimately connected to the impact of multiple feedback mechanisms that evolve over time 9. Because the situation in West Africa is rapidly evolving in time, and attempts at intervention measures are far ranging and also rapidly evolving, understanding how best to control the outbreak, and/or assessing whether or not current attempts to contain the spread are working, poses unique challenges.

In this analysis, we examine current outbreak incidence data for Guinea, Sierra Leone, and Liberia up to September 8, 2014, as estimated by the World Health Organization (WHO) 3 ^,^ 4. Using novel methodology, we assess the impact of adaptive response measures by fitting piecewise exponential curves along the data time series to estimate the evolving rate of exponential rise (or decline) in cases. We then use a Susceptible, Exposed, Infected, Recovered (SEIR) model to estimate the temporal patterns of the effective reproduction number for the outbreak in each country. In the following sections, we describe our sources of data, and the statistical and modeling methods used to estimate the effective reproduction number.

## Methods and Materials

**Data**

As part of the HealthMap project 4, epidemiologists and researchers at the Boston Children’s Hospital have compiled from WHO reports of the time series of confirmed and probable EVD case counts during the 2014 West African outbreak 3. They have made the compilation publicly available online at www.healthmap.org/ebola . The HealthMap data do not include separate information regarding the confirmed and probable cases. For this analysis, the time series of EVD cumulative confirmed and probable cases and deaths in Guinea, Sierra Leone, and Liberia were obtained from HealthMap up to September 4, 2014, and supplemented with more recent data from the WHO website.

The estimated daily average of the new case incidence data for the three countries are shown in Figure 1. Updates to the WHO case counts occur irregularly in time (situation updates have occurred every 2 to 9 days since July 1st ), thus to estimate the daily average incidence data between two updates, we take the new incidence accumulated between updates, and divide by the time between updates.

**Statistical Methods**

Cumulative incidence data are highly inter-correlated from point to point, and thus are inappropriate for fitting with standard least squares or likelihood fitting methods, which have as an underlying assumption that the observations are independent 5 ^,^ 6. Fitting to cumulative incidence data, instead of the time series of number of new cases, can thus lead to dramatically under-estimated confidence intervals on the model parameters, and also potential biases in the fit central values, since points further along the time series (which are nearly 100% correlated) are given too much weight in the fit. In this analysis we thus fit to the time series of newly identified cases for each country.

Using a Negative Binomial likelihood fit to account for over-dispersion in the data 7, for each country we fit piecewise exponential curves along the incidence time series, in which ten contiguous points are fit at a time, and the local exponential rate of rise, \begin{equation*}\small{\rho_{\rm local}}\end{equation*} , is estimated for each. If we have M incidence data points \begin{equation*}\small{Y_i^{\rm obs}}\end{equation*}, recorded between times \begin{equation*}\small{t_i^{\rm min}}\end{equation*} to \begin{equation*}\small{t_i^{\rm max}}\end{equation*}, where i=1,...,M, then the predicted incidence for each point under an exponential rise hypothesis with some rate, \begin{equation*}\small{\rho_{\rm local}}\end{equation*}, is 8:

\begin{equation*}{Y^{\rm pred}_i \sim \int_{t^{\rm min}_i}^{t^{\rm max}_i} dt \exp({\rho_{\rm local} t})}\end{equation*}

which simplifies to

\begin{equation*} Y^{\rm pred}_i \sim \exp(\rho_{\rm local} t^{\rm max}_i) - \exp(\rho_{\rm local} t^{\rm min}_i)}  \;\;\;\;\;\;\;\;\;\;\; \; {\rm  Eqn 1}\end{equation*}

where the \begin{equation*}\small{Y_i^{\rm pred}}\end{equation*} are normalized such that \begin{equation*}\small{\sum_i^M Y_i^{\rm pred} = \sum_i^M Y_i^{\rm obs}}\end{equation*}.

Because of the irregular updates in the data, the time spanned by ten contiguous points ranges from 21 to 34 days. Sensitivity analyses indicate the conclusions of this analysis do not depend on the exact number of contiguous points used for the fits.

We also fit an exponential curve to the entire time series for each country, from July 1st onwards (after July 1st the WHO EVD case count updates for each country became more regular, occurring on average every three days).

**Estimation of the effective reproduction number from the local rates of exponential rise**

The basic reproduction number, \begin{equation*}\small{{\cal{R}}_0}\end{equation*}, of an infectious disease is the average number of secondary cases a case generates during the course of its infectious period, in a completely susceptible population. The basic reproduction number is affected not only by the ease at which an infection is transmitted upon contact between two hosts, but also by the contact rate between members of the population. Because of heterogeneity in susceptibility, infectiousness, and differing social dynamics, the estimates of \begin{equation*}\small{{\cal{R}}_0}\end{equation*} for an infectious disease tend to be different across populations. For example, rural and densely populated urban areas are likely to have substantial differences in contact rates 10. Traditionally \begin{equation*}\small{{\cal{R}}_0}\end{equation*} has been computed under simplifying assumptions, like the existence of a constant contact structure, or under the assumption that interventions like quarantine and isolation are uniformly applied from the very beginning of the epidemic. However, particularly in the case of a disease with high morbidity and mortality, it has been shown that human behaviors adapt to limit spread of the disease 9, thus limiting the usefulness of \begin{equation*}\small{{\cal{R}}_0}\end{equation*} as a means to assess the efficacy of evolving intervention strategies, and to forecast the future progression of the outbreak.

Given these limitations of \begin{equation*}\small{{\cal{R}}_0}\end{equation*}, in this analysis we thus instead assess the time evolution of the effective reproduction number, \begin{equation*}\small{{\cal{R}}_{\rm eff}}\end{equation*}, which is a dynamic estimate of the average number of secondary cases per infectious case in a population composed of both susceptible and non-susceptible individuals during the course of an outbreak. The effective reproduction number is a function of, among other things, the probability of transmission upon contact of the disease, the contact rate within the population, and the number of susceptible individuals in the population, all of which may be time dependent.

In an epidemic, given a short enough period of time, the value of \begin{equation*}\small{{\cal{R}}_{\rm eff}}\end{equation*} during that time period can be considered to be approximately constant. Given a mathematical model for the spread of infectious disease, such as a classic Susceptible, Exposed, Infected, Recovered (SEIR) model, the equations of the mathematical model can be linearized about this temporary “equilibrium”, to determine the predicted local rate of exponential rise of the epidemic curve, \begin{equation*}\small{\rho_{\rm local}}\end{equation*}. For the SEIR model, this is related to \begin{equation*}\small{{\cal{R}}_{\rm eff}}\end{equation*} by

\begin{equation*}{{\cal{R}}_{\rm eff} = (1+\rho_{\rm local}/\gamma)(1+\rho_{\rm local}/\kappa)}\;\;\;\;\;\;\;\;\;\;\; \; {\rm Eqn 2}\end{equation*}

where \begin{equation*}\small{1/\kappa}\end{equation*} and \begin{equation*}\small{1/\gamma}\end{equation*} are the average incubation and infectious periods of the disease, respectively 12.

With estimates of \begin{equation*}\small{\rho_{\rm local}}\end{equation*} from piecewise exponential rise fits to the incidence data from an outbreak (along with estimates of the incubation and infectious periods of the disease), we can thus use Equation 2 to obtain estimates of the temporal behavior of \begin{equation*}\small{{\cal{R}}_{\rm eff}}\end{equation*}, in essence approximating the temporal behavior with a piecewise step-function.

Typically, if the transmissibility and contact rates in the population remain constant, one expects to see the effective reproduction number decrease in time as the fraction of susceptible individuals in the population decreases. However, if \begin{equation*}\small{{\cal{R}}_{\rm eff}}\end{equation*} increases, it can signal that, for instance, contact rates within the population have increased, the virus has become more transmissible, and/or that attempted intervention strategies are actually making the outbreak worse.

While we use an SEIR model and Equation 2 in this analysis to estimate \begin{equation*}\small{{\cal{R}}_{\rm eff}}\end{equation*}, it needs to be stressed that our methodology for estimating the temporal variation in the effective reproduction number from the local rates of exponential rise in incidence can be applied with any mathematical model appropriate to simulate the spread of a particular disease.

## Results

In Figure 1 we show the time series of the recorded daily average incidence data for Guinea, Sierra Leone, and Liberia. Overlaid in green, we show a selection of the piecewise exponential fits along the incidence time series, in which ten contiguous points are fit at a time, and the local exponential rate of rise, \begin{equation*}\small{\rho_{\rm local}}\end{equation*}, is estimated. For clarity of presentation, we only show every third fit. The fit of an exponential to the time series from July 1st onwards is shown overlaid with a red dotted line.

In Figure 2 we show the time series of the estimates of the local exponential rise \begin{equation*}\small{\rho_{\rm local}}\end{equation*} (along with the 95% confidence intervals obtained from the Negative Binomial likelihood fit) for each of the piecewise exponential fits for each country. We also indicate the estimate of the the exponential rise from a fit to the time series from July 1st onwards for each country.

Note that the rates of \begin{equation*}\small{\rho_{\rm local}}\end{equation*} are significantly correlated from point to point.

**Time series of iIncidence of EVD cases in West Africa:**
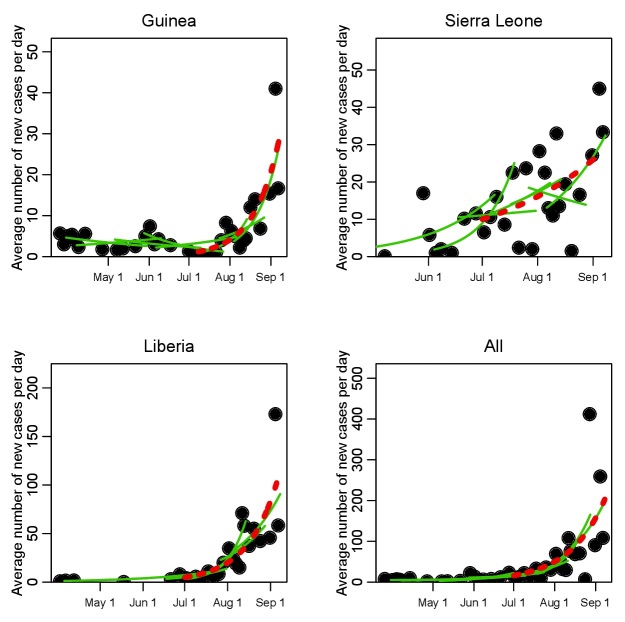
Time series of recorded average number of new EVD cases per day during the initial phase of the 2014 West African outbreak, for Guinea, Sierra Leone, and Liberia (dots). The green lines show a selection of the piecewise exponential fits to the data (not all fits are shown to clarify the presentation); a moving window of groups of 10 contiguous points are taken at a time, and the rate of exponential rise estimated for those 10 points. The results for the estimations of the exponential rise for the full set of piecewise fits are shown in Figure 2. Shown in red is the fitted exponential rise from July 1st onwards.

**Estimated rates of exponential rise from piecewise exponential fits:**
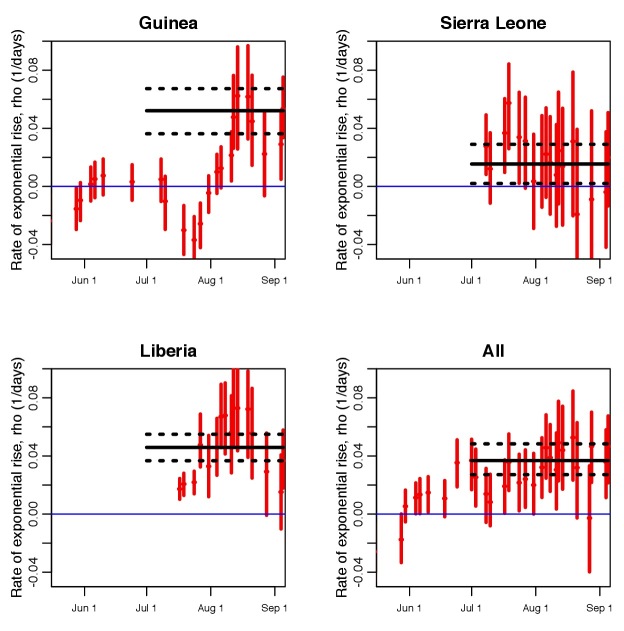
Estimated rates of exponential rise from piecewise exponential fits to the average daily EVD incidence data, as shown in Figure 1; a moving window of groups of 10 contiguous incidence data time series points are taken at a time, and the rate of exponential rise estimated for those 10 points. The dates shown on the x axis are last date in each contiguous set of 10 points, and the vertical error bars denote the 95% confidence interval. The horizontal black line shows the estimated rate of rise of an exponential fit to the incidence time series from July 1st to Sep 8th, with the black dotted lines indicating the 95% confidence interval.

## Discussion

For each country in Figure 2 the solid and dotted black lines denote the central value and 95% CI, respectively, of the rate of exponential rise of case incidence data between July 1st and September 8th . The estimates of the rates of exponential rise between July 1st and September 8th are ρ=0. 052 ± 0. 008, ρ=0. 015 ± 0. 008, and ρ=0. 046 ± 0. 005 for Guinea, Sierra Leone, and Liberia, respectively. The value estimated from the combined data samples is ρ=0. 037 ± 0. 006. The values are statistically consistent for the Guinea and Liberia data (one-sided Z test p = 0. 28). However, the estimate for the Sierra Leone data is significantly lower than the estimates obtained from the Guinea and Liberia data (one-sided Z test p = 0. 0005, and p = 0. 0003, respectively). In Table 1 we show the values of the effective reproduction number, \begin{equation*}\small{{\cal{R}}_{\rm eff}}\end{equation*} , as estimated from Equation 2 under several hypotheses for the incubation and infectious periods (the incubation and infectious periods for this outbreak are poorly known, and have shown wide apparent variation in previous outbreaks 13 ^,^ 14). The reproduction numbers estimated by this analysis are in broad agreement with the reproduction numbers estimated from this and previous outbreaks, which range from approximately 1.3 to 2.7 13 ^,^ 14 ^,^ 22.

**Table 1: Estimates of the effective reproduction number between July 1 to present:** Estimates of the effective reproduction number for the 2014 West African EBV outbreak data, for an SEIR model as obtained from Equation 2 under several hypotheses for the incubation and infectious periods. The estimates and the 95% confidence intervals are derived from the rate of rise of an exponential fit to the new case incidence data for the various countries between July 1st and September 8th (the beginning of July is when the World Health Organization began frequent updates of the new case information for all three countries).

Hypothesized incubation and infectious periods, respectively (days)	Guinea	Liberia	Sierra Leone	All
5 and 5	1.6 [1.4,1.8]	1.5 [1.4,1.6]	1.2 [1.0,1.3]	1.4 [1.3,1.5]
7 and 5	1.7 [1.5,2.0]	1.6 [1.5,1.8]	1.2 [1.0,1.4]	1.5 [1.3,1.7]
10 and 5	1.9 [1.6,2.3]	1.8 [1.6,2.0]	1.2 [1.0,1.5]	1.6 [1.4,1.8]
5 and 7	1.7 [1.5,2.0]	1.6 [1.5,1.8]	1.2 [1.0,1.4]	1.5 [1.3,1.7]
7 and 7	1.9 [1.6,2.2]	1.8 [1.6,1.9]	1.2 [1.0,1.5]	1.6 [1.4,1.8]
10 and 7	2.1 [1.7,2.5]	1.9 [1.7,2.2]	1.3 [1.0,1.6]	1.7 [1.5,2.0]
5 and 10	1.9 [1.6,2.3]	1.8 [1.6,2.0]	1.2 [1.0,1.5]	1.6 [1.4,1.8]
7 and 10	2.1 [1.7,2.5]	1.9 [1.7,2.2]	1.3 [1.0,1.6]	1.7 [1.5,2.0]
10 and 10	2.3 [1.8,2.8]	2.1 [1.9,2.4]	1.3 [1.0,1.7]	1.9 [1.6,2.2]

Two recent papers have attempted to estimate the effect of control measures on the spread of EBV during the West African outbreak. The first (Althaus, 2014) employs a Poisson likelihood fit to the cumulative incidence under the assumption of an SEIR model with control that begins the day the first case appears in the population, and causes the transmission rate to exponentially decay 15. A second recent paper (Fisman *et a*l , 2014) also fits to the cumulative incidence curve, with a model that assumes that the incidence over time is a function of the basic reproduction number, and a control parameter that causes the incidence to decay 16. The Althaus and Fisman *et al* estimates of the reproduction number for the Guinea and Liberia outbreaks are within our corresponding 95% confidence intervals under the same hypotheses for the incubation and infectious periods. However, both analyses predict significantly higher reproduction numbers for the Sierra Leone outbreak. The discrepancy is likely due to the fact that both analyses employ models that assume that control measures are immediately employed and effective; the results thus inherently attribute the low effective reproduction number to a high basic reproduction number being modulated by highly effective control measures. Neither analysis provides a comparison of the model fit results to those of a reduced model where control is excluded, so it is unknown what the estimates for the reproduction number would be if control was not inherently assumed.

We stress again that our analysis makes no assumptions about whether or not control measures have been employed, and/or when they were employed. We simply examine the local rates of exponential rise to estimate how the effective reproduction number appears to be changing in time. We note that up until mid-August, the values of \begin{equation*}\small{\rho_{\rm local}}\end{equation*}for Guinea are significantly below the average central value obtained from the fit the the data from July 1st onwards, and were rising (unless otherwise noted, all subsequent statistical tests are Bonferroni corrected one-sided Z tests, with rejection of the null hypothesis occurring when p < 0.05/k, where k is the number of values being compared). The dip in the two recent Guinea data points compared to the central value is not significant. In addition, the dip in the two recent estimates of \begin{equation*}\small{\rho_{\rm local}}\end{equation*} from the Liberia data are also not significant. However, the estimates of \begin{equation*}\small{\rho_{\rm local}}\end{equation*} for the three earliest points in the Liberia local time series are significantly lower than the central value, and rising. None of the estimates of \begin{equation*}\small{\rho_{\rm local}}\end{equation*} from the Sierra Leone data and combined data for all countries are significantly different than the central value.

It is unclear why the transmission rate of the disease apparently rose for both Guinea and Liberia between mid July to mid August, and why the transmission rate in Sierra Leone is systematically lower, although is important to note that the WHO data are obtained from rudimentary surveillance systems in under-developed countries, under the stress of a rapidly evolving outbreak situation. The temporal patterns we observe may thus partly be due to variations in surveillance during the outbreak, under-reporting, and/or reporting delays. In addition, serial passage of the disease as the outbreak progresses may be leading to increased pathogenicity, and a subsequent increasingly larger rate of increase in case counts . However, it also must be considered that otherwise well intentioned attempts at intervention may in fact be making the situation worse, at least in some regions; in a joint meeting of officials from Guinea, Liberia, and Sierra Leone on Aug 1st , it was announced that *cordons sanitaire* would be implemented, to seal off the villages and regions worst hit by the outbreak, in an attempt to limit its spread outside those areas 17. In addition, at that time Liberia closed all schools and non-essential government offices 18, and two weeks later imposed a military-enforced *cordon sanitaire* on the West Point slum of Monrovia, sparking riots 19. It must be stressed that *cordons sanitaire* do not generally attempt to limit disease spread within the quarantined areas, and the implementation of these measures in West Africa has received criticism due to the fact that the quarantined areas are at risk for crowding, lack of medical and basic services, and poor sanitation, potentially increasing the spread of disease within those areas 20. It was around the period of time that the *cordons sanitaire* were first imposed, and when the outbreak moved to the densely populated cities of Conakry and Monrovia, that the exponential rise in new cases in Guinea and Liberia increased, rather than went down. Again, it is unclear why the rate of exponential rise in Sierra Leone was apparently unaffected by these events.

Based on our estimates of the exponential rise in cases between July 1st to the beginning of September, if this rise is to continue unabated, there will be approximately 4400 new EVD cases in West Africa during the last half of the month of September (95% CI [3000, 6800]), 500, 900, and 3000 of which will be in Sierra Leone, Guinea, and Liberia, respectively.

## Summary

We have presented an analysis where piecewise exponential fits are employed to estimate the local rate of exponential rise in cases in the 2014 West Africa Ebola outbreak. We have shown that these local rates of exponential rise can be used in conjunction with a mathematical model to estimate the temporal changes in the effective reproduction number (in the case of this analysis, we employed an SEIR model). Our analysis indicates that the spread of the disease to densely populated cities, and/or the imposition of *cordons sanitaire * in West Africa may have accelerated the spread of the disease in some regions. Our methodology is novel, and is applicable to any outbreak, and any mathematical disease model.

## Competing Interests

The authors have declared that no competing interests exist.
