# Supplementary material for: Temporal Variations in the Effective Reproduction Number of the 2014 West Africa Ebola Outbreak
Source: PLoS Curr. 2014 Sep 18;6:ecurrents.outbreaks.9e4c4294ec8ce1adad283172b16bc908. [Version 1] doi: 10.1371/currents.outbreaks.9e4c4294ec8ce1adad283172b16bc908 (PMC4169299; doi:10.1371/currents.outbreaks.9e4c4294ec8ce1adad283172b16bc908)
Supplement: Related texts

# Reply

**Published:** 2014-09-19

**Authors:** PLOS Currents

<strong>Correction</strong> The confidence intervals for expected number of cases have been corrected following the discovery of an error by the authors. The correct 95% confidence interval on the expected number of cases by the end of the month is [3000,6800]. This has been updated in the text of the manuscript.
